# Seroepidemiology of *Toxoplasma gondii* Infection in Blood Donors from Western Romania

**DOI:** 10.3390/microorganisms10050973

**Published:** 2022-05-05

**Authors:** Maria Alina Lupu, Rodica Lighezan, Ana Alexandra Paduraru, Angela Dragomir, Radu Pavel, Sebastian Grada, Alin Gabriel Mihu, Sorin Ursoniu, Tudor Rares Olariu

**Affiliations:** 1 Discipline of Parasitology, Department of Infectious Disease, Victor Babes University of Medicine and Pharmacy, 300041 Timisoara, Romania; rlighezan@gmail.com (R.L.); paduraru.ale@gmail.com (A.A.P.); ange.dragomir@gmail.com (A.D.); pavelradu77@gmail.com (R.P.); seba.grada@gmail.com (S.G.); alinmg@yahoo.com (A.G.M.); 2 Center for Diagnosis and Study of Parasitic Diseases, Department of Infectious Disease, Victor Babes University of Medicine and Pharmacy, 300041 Timisoara, Romania; 3 Clinical Laboratory, Institute of Cardiovascular Diseases, 300310 Timisoara, Romania; 4 Regional Blood Transfusion Center, 300737 Timisoara, Romania; 5 Discipline of Epidemiology, Department of Infectious Disease, Victor Babes University of Medicine and Pharmacy, 300041 Timisoara, Romania; 6 Department of Functional Sciences, Center for Translational Research and Systems Medicine, Victor Babes University of Medicine and Pharmacy, 300041 Timisoara, Romania; sursoniu@yahoo.com; 7 Clinical Laboratory, Municipal Clinical Emergency Teaching Hospital, 300254 Timisoara, Romania

**Keywords:** *Toxoplasma gondii*, antibodies, seroprevalence, risk factors, epidemiology, blood donors, Romania

## Abstract

Background: *Toxoplasma gondii* is estimated to infect 33% of blood donors worldwide, and seroprevalence varies widely between countries. We aimed to evaluate, for the first time, the seroprevalence and risk factors associated with *T. gondii* in blood donors from Western Romania. Methods: Serologic testing to demonstrate the presence of *T. gondii* antibodies was conducted in 1347 healthy blood donors. Risk factors for *T. gondii* infection were assessed through an epidemiological questionnaire. Results: The overall prevalence of *T. gondii* antibodies was 45.9%, with a significant age-associated increase (*p* < 0.001) from 32.6% in age group 18–25 years to 67.6% in age group 56–63 years. *T. gondii* seroprevalence decreased with increasing level of education, from 64.3% in individuals who graduated from elementary/middle school to 40.4% in those who graduated from University (*p* < 0.001). The multiple logistic regression analysis revealed that age, level of education and having pets (cats and/or dogs) were significantly associated with *T. gondii* infection. Conclusions: This study brings new and valuable data regarding the seroepidemiology of *T. gondii* infection in Romania. Our findings indicate a high prevalence of *T. gondii* antibodies in blood donors and may serve as a starting point for further epidemiological studies that should lead to implementation of prevention programs for toxoplasmosis.

## 1. Introduction

Toxoplasmosis is a zoonotic infection caused by an obligate intracellular parasite called *Toxoplasma gondii*. This protozoan has a worldwide distribution [1,2]: between 10.0% and 97.4% of the adult population is estimated to be infected [3]. *T. gondii* transmission may involve all three life-cycle stages of the parasite: oocysts (from water, vegetables, or soil), tissue cysts with bradyzoites (from raw or undercooked meat/primary offal), and tachyzoites (from blood products, tissue transplants, or unpasteurized milk) [3,4]. *T. gondii* may be also transmitted from the mother to the fetus by tachyzoites, which can cross the placenta [4]. Most often, the specific route of transmission cannot be established because the vast majority become infected inadvertently [2].

In most immunocompetent patients, the acute phase of the infection is asymptomatic, but clinical signs may appear in congenitally infected children and immunocompromised individuals [2]. Blood donors can impose risk of *T. gondii* infection for the susceptible recipients, such as immunosuppressed individuals and pregnant women [5,6]. Moreover, assessing the seroprevalence of *T. gondii* in blood donors offers important data regarding the prevalence of infection in apparently healthy individuals [7,8] and may indicate the spreading of infection in the general population.

Foroutan-Rad et al. estimated that approximately 33% of blood donors worldwide are infected with *T. gondii*, with the highest global rates on the African continent (46%) and the lowest in Asia (29%) [6]. Seroprevalence varies between countries: 6.26% in China [9], 9.3% in Taiwan [10], 19.66% in India [11], 20.5% in Serbia [7], 25.6% Turkey [12], 36% in Portugal [13], 48.1% in Brasil [14], and 67.92% in Côte d’Ivoire [15].

Previous reports from Western Romania showed a 55.8% prevalence of *T. gondii* antibodies in pregnant women [16], 57.6% among women of childbearing age [17] and 64.8% in the adult population [18]. There is no information regarding the magnitude of infection with *T. gondii* in Romanian blood donors. Therefore, in this study we aimed to evaluate the seroprevalence and risk factors associated with *T. gondii* in blood donors from Western Romania.

## 2. Materials and Methods

### 2.1. Study Design and Study Population

We enrolled 1347 consecutive volunteer healthy blood donors, in the order they presented to the Regional Blood Transfusion Center in Timisoara between 19 November–21 December 2018. Blood donors had to comply with the donation eligibility criteria set by the Romanian Ministry of Health [19]. Individuals with chronic hepatitis, liver cirrhosis, HIV, cancer, schizophrenia, epilepsy, diabetes or anemia were excluded from blood donation following the official blood donation procedure [19].

The software used in the Blood Center provided subjects’ demographic data (age, gender, area of residence) using a code, without their identification. An epidemiological questionnaire was carried out to obtain information regarding the risk factors associated with *T. gondii* infection: level of education (elementary/middle school, high school, University), consumption of raw and/or undercooked meat, contact with soil (gardening and/or agriculture activities), having cat(s) and having dog(s). Study participants were grouped according to their age in 5 age groups: 18–25 years, 26–35 years, 36–45 years, 46–55 years and 56–63 years.

### 2.2. Serological Testing

Serum samples were tested at the Center for Diagnosis and Study of Parasitic Diseases, Victor Babes University of Medicine and Pharmacy, Timisoara, Romania. Pastorex Toxo kit (Bio-Rad, Marnes-la-Coquette, France), a latex particle agglutination assay, was used for simultaneously detection of immunoglobulin G (IgG) and/or immunoglobulin M (IgM) antibodies to *T. gondii*. Pastorex Toxo demonstrated an excellent ability to detect *T. gondii* antibodies in patients with acute and chronic toxoplasmosis [17,18,20]. Testing, quality controls and interpretation of results were based on manufacturer’s criteria.

### 2.3. Statistical Analyses

Statistical analyses were performed using Epi Info Version 7.2 (CDC, Atlanta, GA, USA) and MedCalc for Windows, version 19.4 (MedCalc Software, Ostend, Belgium). Data are presented as number (percentage), mean ± standard deviation (SD), and odds ratio (OR) with 95% confidence interval (CI). For comparison between *T. gondii* positive and negative blood donors, we used 2-tailed Fisher exact test and logistic regression (Wald forward stepwise method) with a *p*-value of <0.05 to represent statistical significance. Multiple logistic regression was performed for those variables that were found to be significantly associated with *T. gondii* infection in univariate analyses.

### 2.4. Ethics and Informed Consent

This study was approved by Victor Babes University of Medicine and Pharmacy Timisoara Ethics Committee (06/16.03.2018). All study participants provided written informed consent.

## 3. Results

The 1347 blood donors enrolled in the study were aged between 18 and 63 years (mean age = 33.6 ± 10.9 years), 755 (56.1%) were males and 979 (72.7%) were residents of urban areas (Table 1).

The overall seroprevalence of *T. gondii* antibodies was 45.9% (95% CI: 43.23–48.55) and tended to increase with age from 32.6% in age group 18–25 years to 67.6% in age group 56–63 years, showing a significant age-associated increase (*p* < 0.001; OR = 1.5; 95% CI: 1.36–1.65) (Table 1).

Compared to blood donors aged 18–25 years, the seroprevalence was significantly higher in individuals aged 26–35 years (42.5%), 36–45 years (56.1%), 46–55 years (61.1%) and 56–63 years (67.6%) (Table 1).

When data were analyzed according to the area of residence, a significantly higher seroprevalence was found in blood donors residing in rural areas (51.1%) compared to those from urban areas (43.9%) (*p* = 0.020) (Table 1).

Both age (*p* < 0.001; OR = 1.5; 95% CI: 1.36–1.65) and area of residence (*p* = 0.036; OR = 1.3; 95% CI: 1.02–1.66) remained statistically significant when they were evaluated using a logistic regression model.

No statistically significant difference was found between rates of infection in females and males (*p* = 0.628) (Table 1).

Of the 592 females included in the study, 493 (83.3%) were aged between 18–45 years (mean age = 28.9 ± 8.2 years) and 72.4% (357/493) of these were residents of urban areas (Table 2). *T. gondii* antibodies were detected in 43.2% (213/493) females of childbearing age, with no significant difference according to area of residence (*p* = 1.00; OR = 0.9; 95% CI: 0.66–1.47) (Table 2). The prevalence of *T. gondii* infection was significantly higher in women aged 46–63 years (63.6%, 63/99) compared to those of childbearing age (*p* < 0.001; OR = 2.3; 95% CI: 1.47–3.60).

*T. gondii* seroprevalence decreased with increasing level of education, from 64.3% in blood donors who graduated from elementary/middle school to 40.4% in those with university (*p* < 0.001; OR = 0.6; 95% CI: 0.55–0.79). Seroprevalence was significantly higher in individuals with elementary/middle school (64.3%, 54/84) compared to those with high school (48.9%, 306/625) (*p* = 0.010; OR = 1.8; 95% CI: 1.17–3.01) and to those with university (40.4%, 258/638) (*p* < 0.001; OR = 2.6; 95% CI: 1.65–4.26) (Table 3).

Consumption of raw and/or undercooked meat was not found to be a risk factor for *T. gondii* infection in blood donors (Table 3). However, individuals who reported contact with soil throughout gardening and/or agriculture activities had higher *T. gondii* seropositivity compared to those who did not confirm having contact with soil (*p* = 0.011; OR = 1.3; 95% CI: 1.07–1.72) (Table 3).

Cat owners (*p* = 0.007; OR = 1.5; 95% CI: 1.12–2.02) and dog owners (*p* < 0.001; OR = 1.5; 95% CI: 1.19–1.97) were both associated with *T. gondii* infection (Table 3). Among those reporting to have any pet (cat and/or dog) *T. gondii* seroprevalence (54.2%, 194/358) was significantly higher compared to those who did not report having any pet (42.9%, 424/989) (*p* < 0.001, OR = 1.5; 95% CI: 1.24–2.01) (Table 3). However, when cat owners and dog owners were combined in a logistic regression model, cat owners were not found to be associated with exposure to *T. gondii* (*p* = 0.386; OR = 1.1; 95% CI: 0.81–1.71), but dog owners remained associated with seropositivity (*p* = 0.040; OR = 1.4; 95% CI: 1.01–1.92).

When age, area of residence, level of education, contact with soil, having cats, having dogs and having any pet (cats and/or dogs) (identified as risk factors for *T. gondii* infection in the univariate analyses), were evaluated using a multiple logistic regression model, only age, level of education and having any pet were found associated with *T. gondii* seropositivity (Table 4).

## 4. Discussion

This is the first report on the seroprevalence and risk factors of *T. gondii* infection in blood donors from Western Romania. The 45.9% seroprevalence found in our study is higher than the 20.5%, 30.6%, 32.1%, 36% and 38.1% seroprevalence reported by European investigators in blood donors from Serbia [7], Bosnia and Herzegovina [21], Czech Republic [22], United Kingdom [23] and Portugal [13], respectively. These differences between countries may be explained by different sample size and various sampling strategies with the study group [7], environmental conditions [3], eating and hygiene habits [2,24] and different assays (with different sensitivities and/or specificities) [6] used to identify the presence of *T. gondii* antibodies.

As previously shown by former authors, we noticed that the prevalence of *T. gondii* antibodies increased with age, and this is a result of a prolonged length of exposure to the parasite [7,12,21,25].

Gender was not associated with *T. gondii* infection in our survey, and this is in accordance with results of previous studies [7,8,10,25].

Our findings showed the presence of *T. gondii* antibodies in serum samples of 43.2% of female blood donors of childbearing age. The seroprevalence was higher than the 7.9% reported in Bosnia and Herzegovina [21] and 22.3% in Taiwan [10]. Sociocultural habits influence the transmission routes of *T. gondii* in a population [21] and may explain the differences observed. Older women were found to be more infected with *T. gondii*, suggesting that females of childbearing age are at greater risk to become infected [7]. However, in Western Romania, we noticed a decrease in *T. gondii* seroprevalence in women of childbearing age from 57.6% in 2008 [17] to 43.2% in 2018. Although in 2008 the study was not conducted in blood donors, the difference in *T. gondii* seropositivity observed after 10 years suggests a possible decline in seroprevalence. This downward trend was recently observed in Serbia (from 85% to 31%) [7] and Italy (from 41.1% to 12.4%) [26], and may be explained by better awareness of *T. gondii* infection especially due to internet sources [7], socioeconomic level increase, improvement in quality of life, and changes in nutritional habits [26].

Educational level has previously been shown as an important risk factor for the occurrence of *T. gondii* infection in pregnant women from Western Romania [16]. In the present study, seropositivity for *T. gondii* decreased with increasing level of education, similar to other reports [8,10,13]. Higher level of education is linked with more knowledge about this infection and its methods of prevention, and a low risk of exposure [16].

Consumption of raw/undercooked meat was not found to be a risk factor for *T. gondii* infection in Romanian blood donors. This confirms our previous findings in Romanian pregnant women [16] and is in agreement with the results recently published by other authors [7,25]. Introduction of modern systems and better hygiene conditions in animal farms [7,26], improved hygiene practices during meat processing [26], increasing use of frozen meat and industrially processed meat products [7] may explain the outcome.

Similar to other reports [10,27], contact with cats was found to be associated with *T. gondii* seropositivity when using an univariate analysis in our study group. However, when logistic regression was performed, contact with cats was no longer identified as a risk factor for *T. gondii*, and this is in agreement with previous findings [7,13]. Interestingly, contact with dogs may increase the risk for toxoplasmosis in our study group. This observation is similar to those reported in previous publications [16,28,29]. Dogs could play an important role in transmission of *T. gondii*, acting as possible mechanical carriers by contaminating their fur with oocysts [28,29,30,31]. In addition, dogs, through feeding on cat feces, can defecate *T. gondii* oocysts, probably after passive gastrointestinal transport [29,30,31,32,33]. In Romanian dogs, *T. gondii* seroprevalence varied from 25% in Central Romania (Cluj-Napoca) [34], to 63% in Southern Romania [35]. Although having pets (cats and/or dogs) was associated with *T. gondii* seropositivity (in univariate analyses) multiple logistic regression analysis did not show having dogs to be an independent risk factor for toxoplasmosis. Having pets (cats and/or dogs) at home was recently found to be a risk factor in Romanian pregnant women [16] and in Chinese pet owners [36]. Therefore, cats and dogs (the most popular pet animals worldwide) may serve as potential sources of infection with *T. gondii* in humans, due to close contact with their owner [37].

Results of our survey indicate that increasing age and lower educational level were also significantly associated with *T. gondii* infection in a multiple logistic regression analysis. Similar findings were previously reported by investigators working in the field. For instance, age persisted as a predictor for *T. gondii* infection in studies conducted in Serbia and Mexico [7,8], and level of education was identified as a risk factor for toxoplasmosis in Portugal when using multivariate regression analyses [13].

Blood donors are usually healthy individuals, with a limited range of age, and prevalence of *T. gondii* infection in blood donors does not reflect the seroprevalence in general population [8]. However, *T. gondii* can be transmitted by blood transfusion from asymptomatic seropositive individuals in early stages of acute infection, adding an extra burden on the global population [6,10]. Moreover, it has been documented the transmission of *T. gondii* by transfusion of leukocytes or platelets, and the possibility of survival of the parasite in citrated blood at 5 °C for more than 50 days [6].

## 5. Conclusions

The present study brings new and valuable data regarding the seroprevalence and basic demographic risk factors for *T. gondii* infection in Romanian blood donors. Our results indicate that the prevalence of *T. gondii* antibodies in this population group is among the highest in Europe. Age, level of education and having pets (cats and/or dogs) were found to be significantly associated with *T. gondii* infection in a multiple logistic regression analysis.

Public health authorities should promote information regarding the epidemiology of *T. gondii* in order to reduce transmission. Our data may serve as a starting point for further studies that should lead to implementation of prevention programs for toxoplasmosis.

## Figures and Tables

**Table 1:** Seroprevalence of *Toxoplasma gondii* infection in blood donors from Western Romania according to age, area of residence and gender.

Variables	No. Tested	Prevalence of *T. gondii* Infection Univariate Analysis		
		N (%)	OR (95% CI)	*p*-Value
Age groups (years)				
18–25	411	134 (32.6)	1 (Ref.)	-
26–35	407	173 (42.5)	1.5 (1.15–2.03)	0.003
36–45	282	158 (56.1)	2.6 (1.93–3.60)	<0.001
46–55	211	129 (61.1)	3.2 (2.30–4.59)	<0.001
56–63	36	24 (67.6)	4.1 (2.01–8.52)	<0.001
Area of residence				
Urban	979	430 (43.9)	1 (Ref.)	-
Rural	368	188 (51.1)	1.3 (1.05–1.70)	0.020
Gender				
Male	755	342 (45.3)	1 (Ref.)	-
Female	592	276 (46.6)	0.9 (0.76–1.18)	0.628
Total	1347	618 (45.9)	-	-

N, number of *T. gondii* seropositive individuals; OR, odds ratio; CI, confidence interval; Ref., reference.

**Table 2:** Seroprevalence of *Toxoplasma gondii* infection in female blood donors from Western Romania aged 18–45 years, according to area of residence.

Variables		No. Tested	Prevalence of *T. gondii* Infection Univariate Analysis		
			N (%)	OR (95% CI)	*p*-Value
Area of residence	Urban	357	154 (43.1)	1 (Ref.)	-
	Rural	136	59 (42.0)	0.9 (0.66–1.47)	1.00
Total		493	213 (43.2)	-	-

N, number of *T. gondii* seropositive individuals; OR, odds ratio; CI, confidence interval; Ref., reference.

**Table 3:** Risk factors for *Toxoplasma gondii* infection in blood donors from Western Romania.

Risk Factors	No. Tested	Prevalence of *T. gondii* Infection Univariate Analysis		
		N (%)	OR (95% CI)	*p*-Value
Level of education				
Elementary/Middle school	84	54 (64.3)	1 (Ref.)	-
High school	625	306 (48.9)	1.8 (1.17–3.01)	0.010
University	638	258 (40.4)	2.6 (1.65–4.26)	<0.001
Consumption of raw and/or undercooked meat				
no	778	371 (47.7)	1 (Ref.)	-
yes	569	247 (43.4)	1.1 (0.96–1.48)	0.121
Contact with soil				
no	949	414 (43.6)	1 (Ref.)	-
yes	398	204 (51.3)	1.3 (1.07–1.72)	0.011
Own cat(s)				
no	1132	501 (44.3)	1 (Ref.)	-
yes	215	117 (54.4)	1.5 (1.12–2.02)	0.007
Own dog(s)				
no	1020	442 (43.3)	1 (Ref.)	-
yes	327	176 (53.8)	1.5 (1.19–1.97)	<0.001
Own any pet: cat(s) and/or dog(s)				
no	989	424 (42.9)	1 (Ref.)	-
yes	358	194 (54.2)	1.5 (1.24–2.01)	<0.001

N, number of *T. gondii* seropositive individuals; OR, odds ratio; CI, confidence interval; Ref., reference.

**Table 4:** Risk factors for *Toxoplasma gondii* infection in multiple logistic regression.

Variables	OR (95% CI)	*p*-Value
Age groups (years)		
18–25	1 (Ref.)	-
26–35	1.7 (1.27–2.28)	<0.001
36–45	2.5 (1.87–3.53)	<0.001
46–55	3.1 (2.15–4.32)	<0.001
56–63	3.8 (1.85–8.00)	<0.001
Level of education		
Elementary/Middle school	1 (Ref.)	-
High school	0.5 (0.36–0.95)	0.031
University	0.4 (0.27–0.72)	0.001
Own any pet: cat(s) and/or dog(s)		
no	1 (Ref.)	-
yes	1.5 (1.17–1.94)	0.001

OR, odds ratio; CI, confidence interval; Ref., reference.

## Data Availability

Not applicable.
